# Alport Syndrome: A Case of Delayed Diagnosis Through Genetic Testing

**DOI:** 10.7759/cureus.95927

**Published:** 2025-11-02

**Authors:** Farid Arman, Niloofar Nobakht, Dianne S Cheung, Huma Kennedy

**Affiliations:** 1 Department of Medicine, Division of Nephrology, David Geffen School of Medicine at UCLA, Los Angeles, USA; 2 Department of Medicine/Endocrinology, Diabetes, and Metabolism, David Geffen School of Medicine at UCLA, Los Angeles, USA; 3 Department of Medicine, Division of Medicine, David Geffen School of Medicine at UCLA, Los Angeles, USA

**Keywords:** alport’s syndrome, asymptomatic hematuria, bilateral hearing loss, genetic test, nephrotic-range proteinuria

## Abstract

Alport syndrome (AS) is a genetic disorder characterized by progressive kidney dysfunction, hearing loss, and ocular abnormalities, with microscopic hematuria often being the initial presenting symptom. Despite being well-recognized among nephrologists, AS has historically been underdiagnosed or misdiagnosed, primarily due to restricted access to genetic testing and its underutilization in clinical practice. The advent of commercial genetic testing has improved diagnostic accuracy. This case report describes a patient with longstanding AS diagnosed late in life through genetic testing.

## Introduction

Alport syndrome (AS) is a genetic disorder affecting the kidneys, ears, and eyes due to mutations in genes encoding type IV collagen (COL4A3, COL4A4, COL4A5), an essential extracellular matrix molecule of the basement membrane in the kidney and other organs [1]. AS has classically been described as X-linked inheritance, with mutations in COL4A5, which account for ~80% of cases. Mutations in COL4A3 and COL4A4 are either inherited in an autosomal recessive (AR) pattern (15%) or an autosomal dominant (AD) pattern (5%) [2].

AS is the second most common Mendelian cause of kidney disease. Estimates of the combined phenotype-based prevalence of XLAS (X-linked AS) and ARAS (autosomal recessive AS) in historical literature vary, ranging from 1:5000 in Utah to 1:17000 in Sweden and 1:53000 in Finland, with the recessive form accounting for around 15% of families. More recently, the analysis of population-based genome sequencing data in GnomAD showed that 1:2320 individuals harbor a predicted pathogenic COL4A5 variant, with the proportion being higher in those with European ancestry (1:1800) compared to those with East Asian (1:2310) or Ashkenazi Jewish (1:4961) ancestry [3,4]. With the advancement of genetic testing, the detection of both the incidence and prevalence of AS is anticipated to increase, enabling more targeted therapeutic strategies for this genetic condition.

## Case presentation

An 80-year-old female with a history of isolated microscopic hematuria for 54 years presented to the nephrology clinic with new-onset hypertension. She had recently transitioned to our healthcare system. Previous evaluations, including a CT urogram with and without intravenous contrast, had revealed bilateral benign simple kidney cysts. A cystoscopy was unremarkable. Proteinuria had been absent until four years before presentation. Her daughter also had isolated microscopic hematuria, although the patient reported no known family history of kidney failure. The labs on presentation are presented in Table 1.

**Table 1:** Select laboratory values on presentation

Variables	Patient values	Normal range
Chemistry		
Sodium	142 mmol/L	135-146 mmol/L
Potassium	4.5 mmol/L	3.6-5.3 mmol/L
Chloride	107 mmol/L	96-106 mmol/L
Total CO_2_	24 mmol/L	20-30 mmol/L
Creatinine	1.7 mg/dL	0.6-1.3 mg/dL
Albumin	3.9 g/dL	3.9-5 g/dL
Urinalysis		
pH	6	05-Aug
Specific gravity	1.013	1.005-1.030
Blood	1+	Negative
Bilirubin	Negative	Negative
Protein	1+	Negative
Red blood cells per high-power field	4	0-11 cells/uL
White blood cells per high-power field	0	0-22 cells/uL
Albumin-to-creatinine ratio	360.3 mcg/mg	<30 mcg/mg
Protein to creatinine ratio	0.6 g/g	0.0-0.4

Serological workup was negative. The patient declined a kidney biopsy. Laboratory results showed a serum creatinine of 1.36 mg/dL in 2017, 1.3 mg/dL in 2022, and 1.8 mg/dL at presentation. Microalbuminuria was 809 mcg at presentation, improving to 300 mcg with angiotensin receptor blocker (ARB) therapy, though the patient initially resisted dose escalation. After diagnosis, she agreed to optimize ARB therapy, with microalbuminuria subsequently at 454 mcg.

Genetic testing via saliva test identified a heterozygous COL4A3 variant (c.338G>T), in an autosomal dominant inheritance pattern predicted to cause a missense mutation (p.Gly1128Val) in exon 39. This previously unreported variant disrupts the Gly-X-Y motif in the collagen triple helix repeat domain, a region where glycine substitutions are associated with AS.

## Discussion

AS is considered rare but has likely been underdiagnosed due to limited access to genetic testing until recently. The patient described here was diagnosed decades after symptom onset, following advanced kidney disease development, highlighting a missed opportunity for earlier intervention to slow disease progression. Hematuria often prompts referral to nephrologists or urologists. After a comprehensive workup, including kidney and bladder imaging, cystoscopy, and serological and urinary tests, many patients receive a diagnosis of “benign isolated hematuria”. The widespread availability of commercial genetic testing has facilitated accurate diagnosis and timely treatment for patients with undiagnosed AS presenting with isolated hematuria. In fact, the most recent guidelines recommend genetic testing as the preferred initial diagnostic test, as it is more accurate than a kidney biopsy [4,5].

Genetic testing for AS should be considered in the following situations: 1. children and young adults (especially females of childbearing age) with isolated persistent glomerular (dysmorphic) haematuria; 2. individuals with persistent haematuria and a family history of either well-documented haematuria or unexplained kidney failure (at least in one first- or second-degree relative) [4]. Genome-wide association studies, particularly in patients with secondary and/or steroid-resistant focal segmental glomerulosclerosis, have shown a strong association between this entity and mutations in the COL4A gene, with a prevalence of up to 20%, which was previously unknown [6].

Although classically X-linked, AS also exhibits autosomal dominant and recessive inheritance patterns, with proportions likely to shift as genetic testing identifies more cases. In X-linked AS, females typically exhibit milder symptoms due to one normal X chromosome, though a subset progresses to kidney failure [7]; autosomal dominant cases, as in this patient, often present with mild hematuria due to heterozygous mutations. Homozygous autosomal recessive cases also have a poor prognosis. Ocular abnormalities and hearing loss are more common in X-linked Alport syndrome as well. Table 2 summarizes the genotype-phenotype correlation in different forms of AS.

**Table 2:** Genotype-phenotype correlation in different patterns of Alport syndrome* ^*^[4] ESRD: end-stage renal disease

Associated complications	X-linked males	X-linked females	Autosomal recessive	Autosomal dominant
Chronic kidney disease and end-stage kidney disease	90% by age 40	15-30% by age 60	62% risk of progression to ESRD	Not reported, but overall rare
Ocular abnormalities	Up to 90%	15%	50-60%	Exceedingly rare
Sensorineural hearing loss	Up to 90%	About 20% by age 60	40-66%	2-13%

Commercial genetic testing has transformed the diagnostic landscape for AS by identifying novel mutations, such as the p.Gly1128Val missense mutation in this case, previously undetected in X-linked-focused testing. This enables diagnosis in patients with atypical presentations or inheritance patterns. Current treatment for AS focuses on early initiation of angiotensin-converting enzyme inhibitors or ARBs to slow renal disease progression [8]. In this case, ARB therapy was delayed until proteinuria reached significant levels, missing an opportunity for earlier renal preservation.

Genetic counseling following diagnosis enabled the patient’s daughter, who had longstanding isolated hematuria, to receive a diagnosis with an autosomal dominant COL4A3 mutation and initiate ARB therapy upon developing stage I hypertension. The patient’s granddaughter, aged 30 years, was recently diagnosed with isolated hematuria, also tested positive for the same COL4A3 mutation. A full ophthalmologic examination should be performed at the time of diagnosis, including dilated fundoscopy. Periodical eye examinations could be performed from the time of diagnosis for XLAS and ARAS (Grade D, expert opinion). Each examination should include a clinical evaluation of all ocular segments with a specific focus on cornea, lens, and retinal alterations. Retinal alterations, including dot-and-fleck retinopathy, temporal retinal thinning, staircase foveopathy, and hypoplastic foveal pit, as well as further retinal alterations, should be documented using optical coherence tomography [4].

It is important to note that the accessibility of genetic testing for the diagnosis of AS has implications for public health: studies have shown that AS is the underlying cause of kidney failure in up to 35% patients with end-stage renal disease of unknown etiology [9]. Given the inherited nature of the disease, identifying the cases promptly can help reduce the risk of progression to end-stage kidney disease in the offspring of these patients, leading to improvements in the health outcomes across multiple generations.

## Conclusions

This report underscores the importance of genetic testing in the evaluation of microscopic hematuria and diagnosing AS. Historically, AS has been underdiagnosed, as a kidney biopsy is often not pursued in cases of isolated hematuria. With the widespread availability of genetic testing, which is more accurate than a kidney biopsy in the diagnosis of AS, this trend is rapidly changing. Early diagnosis enables the timely initiation of kidney-protective therapies and genetic counseling, potentially altering disease progression and informing family planning. Furthermore, the rise in the prevalence of diagnosed cases is expected to lead to more opportunities to investigate and better understand the disease, and potentially design clinical trials evaluating therapeutic strategies. It is imperative to understand the implications of genetic testing for the family and to offer genetic counseling whenever a genetic test is provided to a patient.
